# Supplementary material for: Projected losses of ecosystem services in the US disproportionately affect non-white and lower-income populations
Source: Nat Commun. 2021 Jun 10;12:3511. doi: 10.1038/s41467-021-23905-3 (PMC8192915; doi:10.1038/s41467-021-23905-3)
Supplement: Supplementary file 1 — Supplementary Information [file 41467_2021_23905_MOESM1_ESM.docx]

**Title**

Projected losses of ecosystem services in the US disproportionately affect non-white and lower-income populations

**Authors**

Jesse D. Gourevitch^1,2*^, Aura M. Alonso-Rodríguez^1,2^, Natalia Aristizábal^1,2^, Luz A. de Wit^1,2^_,_ Eva Kinnebrew^1,2^, Caitlin E. Littlefield^2^, Maya Moore^1,3^, Charles C. Nicholson, Aaron J. Schwartz^1,2^, Taylor H. Ricketts^1,2^

**Affiliations**

^1^Gund Institute for Environment, University of Vermont, Burlington, VT

^2^Rubenstein School of Environment and Natural Resources, University of Vermont, Burlington, VT

^3^Food Systems Program, University of Vermont, Burlington, VT

* Corresponding author

**Email:** [Jesse.Gourevitch@uvm.edu](mailto:Jesse.Gourevitch@uvm.edu)

**Keywords**

ecosystem services, equity, land cover, population, United States

**Supplemental Information**

**Supplementary Table 1.** Regional classifications by state.

| **Regions** | **States** |
| --- | --- |
| Midwest | Illinois, Indiana, Iowa, Kansas, Michigan, Minnesota, Missouri, Nebraska, North Dakota, Ohio, South Dakota, Wisconsin |
| Northeast | Connecticut, Maine, Massachusetts, New Hampshire, New Jersey, New York, Pennsylvania, Rhode Island, Vermont |
| South | Alabama, Arkansas, Delaware, Florida, Georgia, Kentucky, Louisiana, Maryland, Mississippi, North Carolina, Oklahoma, South Carolina, Tennessee, Texas, Virginia, West Virginia |
| West | Arizona, California, Colorado, Idaho, Montana, Nevada, New Mexico, Oregon, Utah, Washington, Wyoming |

**Supplementary Table 2.** Emission factors by land cover class. All units are in Kg ⋅ Ha^-1^ ⋅ yr^-1^.

|  | VOC | NH_3_ | NO_x_ | SO_2_ |
| --- | --- | --- | --- | --- |
| Urban | N/A | N/A | 7.02E-05 | 1.34E-03 |
| Forest | 3.91E-05 | N/A | N/A | N/A |
| Grassland | 6.44E-06 | N/A | N/A | N/A |
| Cropland | 2.33E-07 | 5.00E-04 | 1.00E-04 | N/A |
| Wetland | N/A | N/A | N/A | N/A |

**Supplementary Figure 1 – Scenario A1B.** Maps of changes in ES supply and demand between 2020 and 2100 at the county-level across the conterminous US. These maps only show results for *scenario A1*. Counties where supply or demand change between -5% and 5% are plotted with lower hues. Purple areas outlined in red indicate counties where supply and demand mismatches are expected to occur.

**Supplementary Figure 1 – Scenario A2.** Maps of changes in ES supply and demand between 2020 and 2100 at the county-level across the conterminous US. These maps only show results for *scenario A2* and are identical to the maps in Figure 3. Counties where supply or demand change between -5% and 5% are plotted with lower hues. Purple areas outlined in red indicate counties where supply and demand mismatches are expected to occur.

**Supplementary Figure 1 – Scenario B1.** Maps of changes in ES supply and demand between 2020 and 2100 at the county-level across the conterminous US. These maps only show results for *scenario B1*. Counties where supply or demand change between -5% and 5% are plotted with lower hues. Purple areas outlined in red indicate counties where supply and demand mismatches are expected to occur.

**Supplementary Figure 1 – Scenario B2.** Maps of changes in ES supply and demand between 2020 and 2100 at the county-level across the conterminous US. These maps only show results for *scenario B2*. Counties where supply or demand change between -5% and 5% are plotted with lower hues. Purple areas outlined in red indicate counties where supply and demand mismatches are expected to occur.

**Supplementary Figure 2.** Scatterplot comparing the percentage of white farm operators (y-axis) with the percentage of white people in the general population (x-axis), by county. On average, farm operators are 15% more white than the general population of the county. The grey line indicators the one-to-one line, whereby Y = X. The red dashed line indicators a fitted OLS model. Farm operator demographic data were acquired from the USDA NASS 2012 Census. General population demographic data were acquired from the US Census.


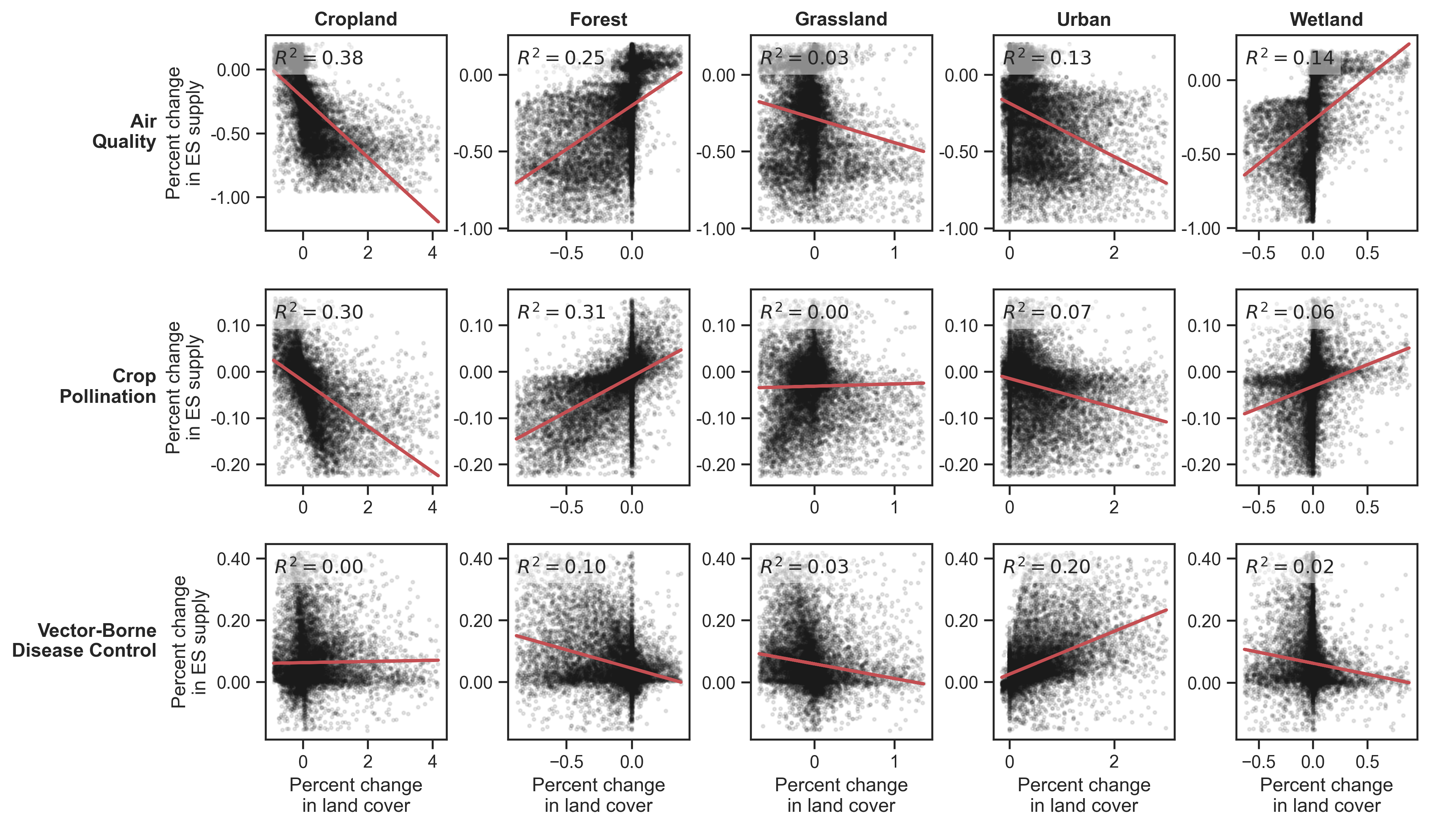


**Supplementary Figure 3.** Associations between change in ES supply and change in land cover between 2020 and 2100. All four scenarios are plotted on each panel, such that each county is plotted four times. The data were fitted using a simple linear regression, as shown by the red trendline. The R^2^ for the linear regression is shown in the upper-left corner of each plot.

**
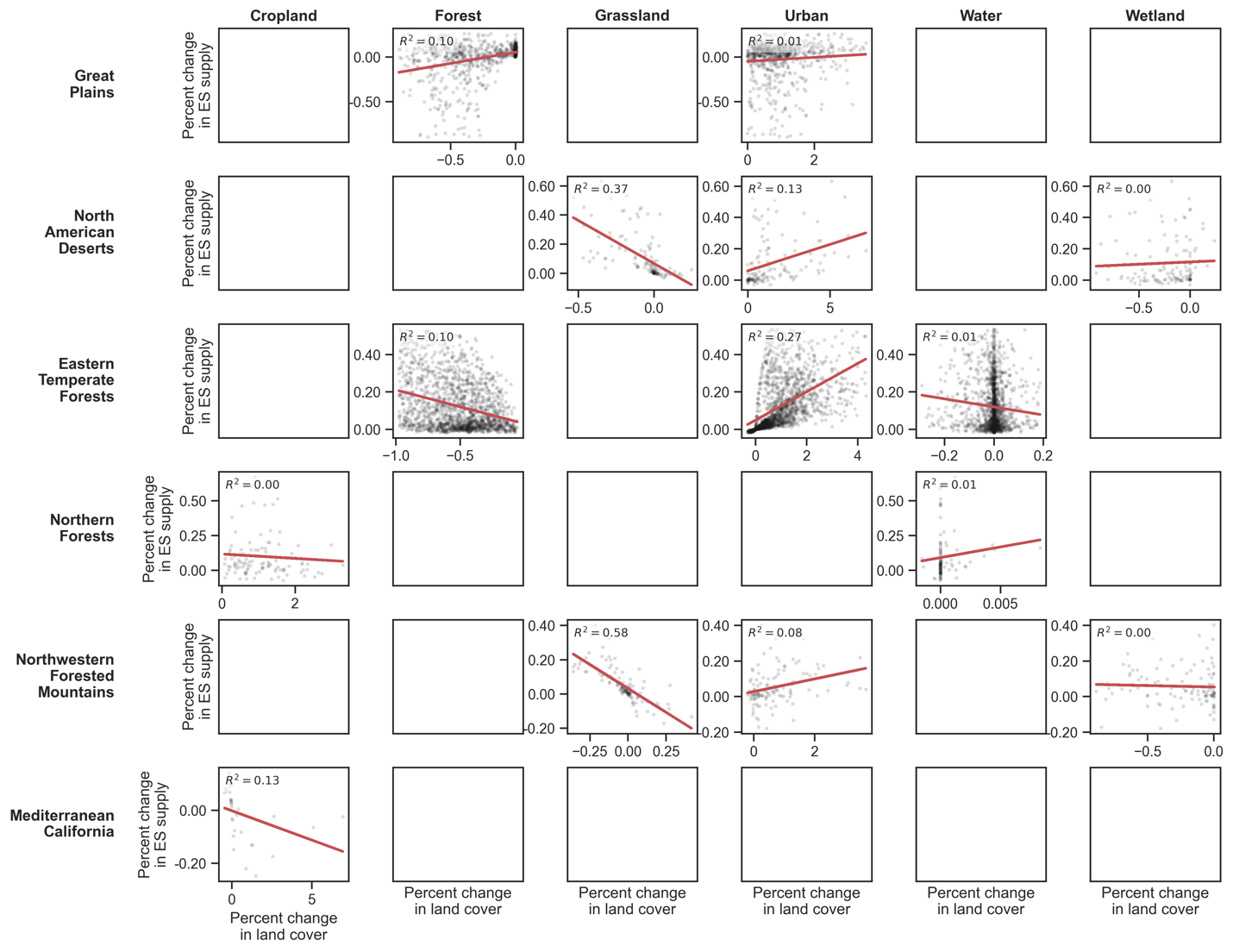
**

**Supplementary Figure 4.** Associations between change in avoided risk of West Nile virus (WNV) and change in land cover between 2020 and 2100 by ecoregion. Blank plots indicate land cover classes that were not included as predictors in the WNV model. All four scenarios are plotted, such that each county is plotted four times. The data were fitted using a simple linear regression, as shown by the red trendline. The R^2^ for the linear regression is shown in the upper-left corner of each plot.
