# Supplementary material for: Projected losses of ecosystem services in the US disproportionately affect non-white and lower-income populations
Source: Nat Commun. 2021 Jun 10;12:3511. doi: 10.1038/s41467-021-23905-3 (PMC8192915; doi:10.1038/s41467-021-23905-3)
Supplement: Supplementary file 3 — Reporting Summary [file 41467_2021_23905_MOESM3_ESM.pdf]

nature research

Corresponding author(s): Jesse D. Gourevitch

Last updated by author(s): May 3, 2021

Reporting Summary

Nature Research wishes to improve the reproducibility of the work that we publish. This form provides structure for consistency and transparency in reporting. For further information on Nature Research policies, see our Editorial Policies and the Editorial Policy Checklist.

Statistics

For all statistical analyses, confirm that the following items are present in the figure legend, table legend, main text, or Methods section.

n/a	Confirmed	
☐	☒	The exact sample size (n) for each experimental group/condition, given as a discrete number and unit of measurement
☒	☐	A statement on whether measurements were taken from distinct samples or whether the same sample was measured repeatedly
☒	☐	The statistical test(s) used AND whether they are one- or two-sided<br>*Only common tests should be described solely by name; describe more complex techniques in the Methods section.*
☐	☒	A description of all covariates tested
☐	☒	A description of any assumptions or corrections, such as tests of normality and adjustment for multiple comparisons
☒	☐	A full description of the statistical parameters including central tendency (e.g. means) or other basic estimates (e.g. regression coefficient) AND variation (e.g. standard deviation) or associated estimates of uncertainty (e.g. confidence intervals)
☒	☐	For null hypothesis testing, the test statistic (e.g. F, t, r) with confidence intervals, effect sizes, degrees of freedom and P value noted<br>*Give P values as exact values whenever suitable.*
☒	☐	For Bayesian analysis, information on the choice of priors and Markov chain Monte Carlo settings
☒	☐	For hierarchical and complex designs, identification of the appropriate level for tests and full reporting of outcomes
☐	☒	Estimates of effect sizes (e.g. Cohen's d, Pearson's r), indicating how they were calculated

Our web collection on statistics for biologists contains articles on many of the points above.

Software and code

Policy information about availability of computer code

Data collection	No software was used in data collection, since all data inputs are derived from publicly-available dataset.
Data analysis	InMAP v1.6.1; glmmulti & boot packages in R v3.6.1

For manuscripts utilizing custom algorithms or software that are central to the research but not yet described in published literature, software must be made available to editors and reviewers. We strongly encourage code deposition in a community repository (e.g. GitHub). See the Nature Research guidelines for submitting code & software for further information.

Data

Policy information about availability of data

All manuscripts must include a data availability statement. This statement should provide the following information, where applicable:

- Accession codes, unique identifiers, or web links for publicly available datasets
- A list of figures that have associated raw data
- A description of any restrictions on data availability

The output data that support the findings of this study are available in a FigShare repository with the following identifier: 10.6084/m9.figshare.13622774

Field-specific reporting

Ecological, evolutionary & environmental sciences study design

All studies must disclose on these points even when the disclosure is negative.

Study description	We use land cover and population projections to model potential shifts in the supply, demand, and benefits of the following ES: climate regulation, provision of clean air, protection against a vector-borne disease (West Nile virus), and crop pollination.
Research sample	United States Geological Survey FOREcasting SCEnarios of Land-Use Change (FORE-SCE) dataset; United States Environmental Protection Agency (US EPA) Integrated Climate and Land Use Scenarios (ICLUS) dataset
Sampling strategy	Sampling strategy is not applicable because all counties in the conterminous United States were used in our sample.
Data collection	Data downloaded using Google Chrome by Jesse D. Gourevitch in January 2020
Timing and spatial scale	Timing: Years 2020 and 2100; Spatial Scale: Conterminous United States
Data exclusions	No data were excluded from the analysis.
Reproducibility	All code will be made available upon request.
Randomization	Randomization is not applicable because all counties in the conterminous United States were used in our sample.
Blinding	Blinding was not relevant to our study because our methods are entirely based on computational models.

Did the study involve field work? ☐ Yes ☒ No

Reporting for specific materials, systems and methods

We require information from authors about some types of materials, experimental systems and methods used in many studies. Here, indicate whether each material, system or method listed is relevant to your study. If you are not sure if a list item applies to your research, read the appropriate section before selecting a response.

Materials & experimental systems

n/a	Involved in the study
☒	☐ Antibodies
☒	☐ Eukaryotic cell lines
☒	☐ Palaeontology and archaeology
☒	☐ Animals and other organisms
☒	☐ Human research participants
☒	☐ Clinical data
☒	☐ Dual use research of concern

Methods

n/a	Involved in the study
☒	☐ ChIP-seq
☒	☐ Flow cytometry
☒	☐ MRI-based neuroimaging
